# Hepatitis C in Liver Allograft Recipients: Utility of One-Year Post-Transplantation Biopsy as an Indicator of Antiviral Therapy

**DOI:** 10.14740/gr694w

**Published:** 2015-12-31

**Authors:** Shahid Habib, Shahid Malik, Bo Fu, Joyce Chang, Michael Nalesnik, Abhinav Humar, Obaid S. Shaikh

**Affiliations:** a Division of Gastroenterology & Hepatology, Liver Research Institute, University of Arizona, Tucson, AZ, USA; b Division of Gastroenterology, Hepatology & Nutrition, University of Pittsburgh School of Medicine, Pittsburgh, PA, USA; c Department of Biostatistics, Graduate School of Public Health, University of Pittsburgh, Pittsburgh, PA, USA; d Division of General Internal Medicine, University of Pittsburgh School of Medicine, Pittsburgh, PA, USA; e Division of Transplantation Pathology, University of Pittsburgh School of Medicine, Pittsburgh, PA, USA; f Division of Transplantation Surgery, University of Pittsburgh School of Medicine, Pittsburgh, PA, USA

**Keywords:** Antiviral agent, Hepatitis C, Immunosuppression, Liver fibrosis, Transplantation

## Abstract

**Background:**

All-oral interferon-free regimens for hepatitis C viral (HCV) infection are highly efficacious; however, high cost is a barrier to applicability. Liver allograft recipients are particularly likely to benefit from therapy as HCV often leads to graft dysfunction and loss. In this study, we aimed to establish the utility of allograft biopsy at 1 year post-transplant as an indicator of treatment.

**Methods and Results:**

Among 252 liver recipients enrolled, 136 (54%) developed severe disease (fibrosing cholestatic hepatitis (FCH) or fibrosis stage ≥ 2 at 1 year post-transplant). Multivariable analysis revealed younger recipient age and female gender, older donor age and T cell depletive therapy to be independent predictors of severe disease. Recipients with severe disease had higher rate of further graft loss compared to those with mild disease. Patients with mild disease and sustained virologic response (SVR) had the best survival rate, whereas those with severe disease and viremia had the worst survival (96% versus 63% at 5 years).

**Conclusion:**

In conclusion, allograft biopsy at 1 year helps identify recipients at high risk of further graft dysfunction and loss. In view of high cost of therapy, treatment should be preferably directed to high-risk patients including those with FCH or fibrosis stage ≥ 2 by 1 year post-transplant.

## Introduction

Among adult patients who undergo liver transplantation in the United States, 23% have hepatitis C virus (HCV) infection (Organ Procurement and Transplantation Network; http://optn.transplant.hrsa.gov; accessed July 2014). Hepatitis C is notable as a cause of morbidity in liver transplant recipients that results from universal recurrence of infection and an accelerated disease course. Thus, HCV-positive recipients tend to have inferior graft and patient survival compared to HCV-negative recipients [1]. The severity of post-transplant hepatitis C has a wide spectrum that ranges from minimal inflammatory activity without fibrosis to progressive graft fibrosis and graft failure to rapid and early graft failure from fibrosing cholestatic hepatitis (FCH) [2]. Certain features have been identified to be associated with inferior outcomes. Among them donor age, pre-transplant viral load, acute rejection episodes and use of bolus corticosteroids and T cell depletive therapy have been found to be particularly influential [3, 4].

Highly effective interferon-free regimens, incorporating NS5A, NS5B and protease inhibitors with or without ribavirin, are now the standard of care for HCV genotypes 1 and 4. For genotypes 2 and 3, sofosbuvir, an NS5B inhibitor, with ribavirin is recommended. The safety and efficacy of interferon-free regimens in transplant recipients remain under investigation [5]. Current regimens have little interaction with calcineurin inhibitors, which was prominent with first-generation protease inhibitors [6]. However, as ribavirin is likely needed in this difficult to treat, immunosuppressed population, anemia will remain a consideration during therapy [7]. The use of all-oral regimens has been hampered by prohibitive cost particularly in low income countries [8, 9]. Transplant recipients with HCV form a special population where viral eradication is highly desirable in view of accelerated disease course and risk of graft failure. Considering limited resources in many parts of the world, it will be prudent to prioritize therapy to recipients with severe disease and/or to those likely to develop severe disease. Patients with mild disease could be followed closely and treated when more affordable regimens become available or if their disease progresses. One approach is to base treatment decisions on protocol allograft biopsies as liver pathologies at 4 months and at 1 year of transplantation have been shown to differentiate subsequent slow or rapid progression of HCV disease [10, 11].

The aim of our study was to establish the utility of allograft biopsy at 1 year post-transplant as a determinant of further progression of disease. We hypothesized that the development of FCH or fibrosis stage ≥ 2 at 1 year post-transplant was associated with rapid progression of disease leading to early graft failure. Such patients would therefore benefit from effective antiviral treatment causing viral clearance. Our secondary goals were to determine factors that influenced disease progression and to determine the impact of sustained virologic response (SVR) to antiviral therapy on long-term allograft and patient survival.

## Patients and Methods

### Patient enrollment

We enrolled adult, HCV-positive liver transplant recipients in a prospective follow-up cohort study. The study required subjects to be anti-HCV positive, have detectable HCV RNA and hepatitis B surface antigen (HBsAg) and antibody to human immunodeficiency virus (HIV) negative. Recipients who died within 1 month of transplantation were excluded from the current study. Once a patient was identified to be a suitable subject, a research coordinator introduced the study to the patient and provided details of study participation. Subjects were recruited in the clinic that provided longitudinal care to liver transplant patients. Follow-up visits were scheduled at 3 - 6 monthly intervals or more frequently if indicated. All participants signed an Institutional Review Board (IRB) approved consent form.

### Patient evaluation

At the initial evaluation, a complete history and examination were obtained. In addition, laboratory, radiologic and pathologic features were noted, and Child-Pugh and model for end-stage liver disease (MELD) scores were calculated. Donor and operative variables studied were age, gender, race, live or deceased, mismatch for gender, race and blood group, cold and warm ischemia time, operative technique and transfusion requirements. Immunosuppressive regimen, post-transplant course and complications were also recorded. Protocol allograft biopsies were obtained at 1 year following transplantation and every 3 years, subsequently. Additional biopsies were obtained as clinically indicated. All biopsies were read by the study pathologist. Hepatic inflammatory activity and fibrosis were graded and staged according to the Ishak’s histology activity index [12], and graft rejection was graded according to the Banff schema [10]. If a graft was lost within 4 weeks of transplantation, we included the second graft instead. Ten milliliter of whole blood was obtained at each visit and separated serum was stored at -80 °C for later studies.

### Anti-HCV therapy

We initiated antiviral therapy if a patient developed FCH or if fibrosis stage was ≥ 2 at 1 year post-transplant. Treatment was otherwise deferred until criteria were met on subsequent evaluations. The decision to treat patients with stage ≥ 2 fibrosis was based on our understanding that such patients had high likelihood of progression to graft failure and death [11]. Conversely, recipients with lesser degree of fibrosis had low risk of disease progression and graft failure. In low-risk group, treatment was offered to those who requested treatment, regardless of histologic severity. Patients noted to have negative HCV RNA 6 months after discontinuation of treatment were considered to have SVR. Patients who received antiviral treatment and either did not respond or relapsed after discontinuation of therapy were considered viremic. The latter group also included those who did not receive antiviral therapy.

### Study endpoints

The primary endpoint was cumulative graft and patient survival among patients alive at 1 year post-transplant. Recipients categorized to have mild (stage 0 - 1 fibrosis) versus severe (stage 2 - 6 fibrosis or FCH) disease were compared, and factors predictive of graft loss were determined. In addition, we determined factors that predicted development of severe disease by 1 year post-transplant.

### Statistical analysis

We evaluated recipient, donor and operative variables at the time of transplantation and compared patients categorized to have mild versus severe disease at 1 year post-transplant. Continuous variables were assessed by the Student’s *t*-test and categorical variables by Chi-square test with Yates continuity correction if applicable. Logistic regression analysis was used to determine factors predictive of disease severity as described. Cumulative survival was determined by Kaplan-Meier analysis and the two severity groups were compared by log-rank test. Factors predictive of long-term survival were determined by Cox proportional hazards modeling. All analyses were performed using SAS^TM^ 9.2 and R^TM^ 2.12.1.

## Results

### Study cohort

**Figure 1:**
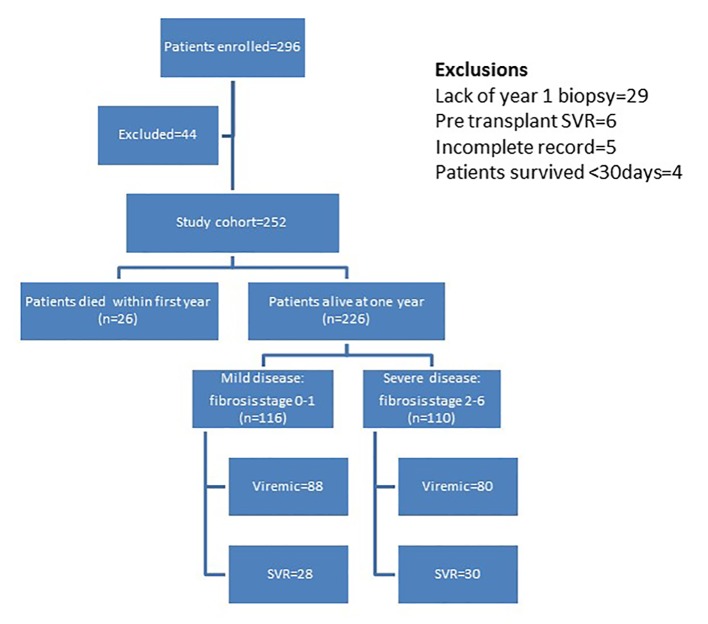
Distribution of patients in study cohort.

Figure 1 outlines the distribution of the study cohort. Two hundred ninety-six liver transplant recipients were enrolled over a period of 8 years (2002 - 2009). Forty-four recipients were excluded from this study for the following reasons: 29 did not have 1 year allograft biopsy, six were HCV RNA negative pre- and post-transplant, five had incomplete information and four survived < 30 days post-transplant; thus, 252 recipients were studied. Based on 1 year post-transplant histologic evaluation and outcome, 116 recipients were categorized to have mild disease (fibrosis stage 0 - 1) and 136 were categorized to have severe disease (110 with fibrosis stage 2 - 6, 26 with post-transplant survival of < 1 year). Patients were followed for a mean follow-up of 5.6 years (SD ± 2.8).

The mean age of the cohort was 51 years and 77% were men. More than 90% were Caucasian, they largely had Child’s B or C disease and their mean MELD score at transplantation was 16. Almost one-third had co-morbidities including obesity (34%), hypertension (34%), diabetes mellitus (29%) and renal failure (15%). Known or incidental hepatocellular carcinoma was noted in 27% of the recipients. Among patients with available HCV RNA level, 32% had viral load of > 850,000 international units per mL (IU/mL) and 84% had HCV genotype 1. Majority were seropositive for cytomegalovirus (CMV) and Epstein-Barr virus. Fourteen percent of donors were anti-HCV positive, 11% were antibody to hepatitis B core antigen (anti-HBc) positive and 62% were seropositive for CMV. Tacrolimus was the primary immunosuppressant and in addition, one-quarter received mycophenolate. Most patients were given corticosteroids, typically intravenous methylprednisone followed by oral prednisone that was generally weaned off within 6 months. About 15% received lymphocyte depletive therapy with either anti-thymocyte globulin or alemtuzumab, during the peri-operative and/or immediate post-operative phase.

### Factors related to severity of disease during first year of transplantation

#### Univariable analysis

One hundred thirty-six patients (54%) developed severe disease within first year of transplantation (Tables 1-5). Factors significantly associated with severe disease included female gender, high white cell count and older donor age; however, no particular age threshold was noted. We examined several operative features including cold and warm ischemia time and the use of blood products (red blood cells, platelets and fresh frozen plasma). In addition, we looked at operative techniques, choledocho-choledocho anastomosis versus choledocho-jejunostomy, veno-venous bypass, graft placement in a piggyback fashion with inferior venacava preservation versus standard implantation and length of hospital stay. None of those features was significantly different among the two groups. Patients with severe disease were more likely to have received T cell depletive therapy, had a higher likelihood of acute rejection episodes and of treatment with bolus corticosteroids. In addition, they received higher cumulative doses of tacrolimus per unit of time. About 5% develop changes consistent with chronic rejection.

**Table 1:** Demographics and Baseline Clinical Features

	Mild disease (n = 116)	Severe disease (n = 136)	P value
Age (years)	52 ± 7	51 ± 6	0.06
Gender (male)	98 (84%)	96 (71%)	0.009
Caucasian	107 (92%)	124 (91%)	0.76
Child-Pugh status			0.14
Class A	12 (10%)	6 (4%)	
Class B	69 (59%)	80 (59%)	
Class C	35 (30%)	50 (37%)	
Child-Pugh score	9 ± 1.8	9 ± 1.6	0.12
MELD score	16 ± 7	16 ± 8	0.87
Blood group			0.62
A	40 (34%)	56 (41%)	
B	11 (9%)	14 (10%)	
AB	9 (8%)	7 (5%)	
O	56 (48%)	59 (43%)	
Co-morbidities			
Diabetes mellitus	35 (30%)	37 (27%)	0.60
Hypertension	43 (37%)	43 (32%)	0.36
BMI (kg/m^2^)	29 ± 5	28 ± 5	0.12
Obesity (BMI ≥ 30)	44 (38%)	43 (32%)	0.29
Renal Failure	16 (14%)	22 (16%)	0.60
Hepatocellular carcinoma	32 (28%)	37 (27%)	0.95

All values are shown as mean ± SD or proportion as appropriate. Comparison by two-tailed *t*-test or Chi-square test as appropriate. n: number. SD: standard deviation.

**Table 2:** Baseline Laboratory Features

	Mild disease (n = 116)	Severe disease (n = 136)	P value
Hemoglobin (g/dL)	12.0 ± 2.1	11.7 ± 2.1	0.20
WBC (× 10^3^/L)	5.0 ± 2.7	5.9 ± 4.0	0.04
Platelets (× 10^3^/L)	67 ± 40	74 ± 50	0.25
Bilirubin (mg/dL)	4.1 ± 6.2	4.6 ± 7.4	0.55
ALT (IU/L)	131 ± 411	152 ± 420	0.69
AST (IU/L)	334 ± 1926	255 ± 914	0.67
ALP (IU/L)	166 ± 106	164 ± 88	0.87
γGTP (IU/L)	97 ± 116	95 ± 97	0.89
Albumin (g/dL)	2.9 ± 0.5	2.8 ± 0.6	0.29
INR	1.4 ± 0.4	1.4 ± 0.5	0.95
Creatinine (mg/dL)	1.3 ± 1.3	1.4 ± 1.4	0.77
HCV RNA (× 10^6^ IU/mL)*	1.02 ± 3.4	2.0 ± 6.4	0.16
HCV genotype 1**	89 (81%)	111 (87%)	0.22
Anti-CMV, IgG (IU/L)	105 ± 122	102 ± 126	0.86
Anti-CMV, IgG-positive***	81 (70%)	81 (60%)	0.09
Anti-EBV, IgG (IU/L)	251 ± 222	242 ± 196	0.75
Anti-EBV, IgG-positive****	79 (71%)	101 (77%)	0.24

All values are shown as mean ± SD or proportion as appropriate. Comparison by two-tailed *t*-test or Chi-square test as appropriate. *n = 209. **n = 238; data unavailable in 14 patients (mild disease: 5, severe disease: 9). ***Cytomegalovirus antibody (positive: > 4 IU/L). ****Epstein-Barr viral capsid antigen antibody (positive: > 20 IU/L); n = 243. n: number. SD: standard deviation; IU: international units.

**Table 3:** Donor Features

	Mild disease (n = 116)	Severe disease (n = 136)	P value
Age (years)	41 ± 17	45 ± 16	0.026
Males	68 (59%)	78 (57%)	0.84
Caucasian	96 (83%)	120 (88%)	0.21
Living donor	9 (8%)	14 (10%)	0.49
Anti-HCV positive*	13 (11%)	22 (17%)	0.25
Anti-HBc positive**	13 (12%)	14 (11%)	0.86
Anti-CMV IgG positive***	70 (63%)	82 (61%)	0.76
Donor biopsy	61 (53%)	82 (60%)	0.22
Blood group			0.83
A	39 (34%)	52 (38%)	
B	13 (11%)	13 (10%)	
AB	7 (6%)	6 (4%)	
O	57 (49%)	65 (48%)	
Donor recipient mismatch			
Gender	48 (41%)	58 (43%)	0.84
Racial	24 (21%)	26 (19%)	0.75
ABO blood group	5 (4%)	7 (5%)	0.76

All values are shown as mean ± SD or proportion as appropriate. Comparison by two-tailed *t*-test or Chi-square test as appropriate. *n = 247. **n = 243. ***n = 245. n: number. SD: standard deviation; anti-HCV: antibody to hepatitis C virus; anti-HBc: antibody to hepatitis B core antigen; CMV: cytomegalovirus.

**Table 4:** Operative Features

	Mild disease (n = 116)	Severe disease (n = 136)	P value
Cold ischemia time (h)	9.8 ± 3.4	9.9 ± 3.9	0.82
Warm ischemia time (min)	34 ± 11	33 ± 10	0.24
Red cell transfusion (unit)*	7.5 ± 6.7	8.5 ± 7.5	0.28
Platelets (unit)	6.2 ± 8.1	7.7 ± 9.4	0.20
Fresh frozen plasma (unit)*	6.4 ± 6.6	7.6 ± 7.3	0.20
Piggyback graft placement	98 (84%)	117 (86%)	0.73
Duct-duct anastomosis	110 (95%)	129 (95%)	1.0
T-tube placement	77 (66%)	96 (71%)	0.47
Veno-venous bypass	81 (70%)	94 (69%)	0.90
Length of stay post-transplant (days)	19 ± 19	21 ± 23	0.64

All values are shown as mean ± SD or proportion as appropriate. Comparison by two-tailed *t*-test or Chi-square test as appropriate. *n = 246. n: number. SD: standard deviation.

**Table 5:** Immunosuppression and Antiviral Therapy

	Mild disease (n = 116)	Severe disease (n = 136)	P value
Corticosteroids	107 (92%)	121 (89%)	0.38
Anti-T cell antibody*	10 (9%)	27 (20%)	0.012
Mycophenolate mofetil	29 (25%)	33 (24%)	0.89
Sirolimus	1 (1%)	4 (3%)	0.24
Acute rejection	33 (28%)	60 (44%)	0.01
Acute rejection episodes	0.4 ± 0.7	0.6 ± 0.8	0.034
ACR: steroid boluses	29 (25%)	50 (37%)	0.045
Chronic rejection	4 (3%)	9 (7%)	0.26
Cumulative steroid dose (g)**	154 ± 780	214 ± 781	0.54
Cumulative tacrolimus dose (mg/year)	467 ± 577	686 ± 996	0.038
Interferon therapy***	79 (68%)	99 (73%)	0.41
Interferon responders	28 (24%)	31 (23%)	0.8

All values are shown as mean ± SD or proportion as appropriate. Comparison by two-tailed *t*-test or Chi-square test as appropriate. n: number. SD: standard deviation. *Perioperative with either antithymocyte globulin or alemtuzumab. **During the first post-operative year and hydrocortisone equivalent. ***Post-transplant treatment with pegylated interferon-α2b or pegylated interferon-α2a with ribavirin.

#### Multivariable analysis

We selected factors previously recognized to influence disease severity and those found significant on univariable analysis for forced entry into logistic regression (Table 6). After multiple iterations, we reached the most efficient model that included four variables. The assumptions of linearity of logit and lack of multicollinearity were tested and were noted to be fulfilled. Younger recipient age, female gender, older donor age and use of anti-T cell antibody were independently predictive of severe disease. Female gender had the highest effect size with an odds ratio of 2.6. The model was significant and explained 7-12% of variability in the likelihood of development of severe disease as indicated by the R^2^ values.

**Table 6:** Predictors of Disease Severity at 1 Year Post-Transplant

Variables included	B ± SE	Odds ratio	95% CI	P value
Recipient age	-0.055 ± 0.02	0.95	0.91 - 0.98	0.007
Female gender	0.96 ± 0.34	2.6	1.33 - 5.07	0.005
Donor age	0.02 ± 0.01	1.02	1.0 - 1.04	0.021
Anti-T cell antibody	0.85 ± 0.41	0.43	0.19 - 0.95	0.037
Constant	2.7 ± 1.12	14.88		0.016

Logistic regression analysis. R^2^ = 0.07 (Hosmer & Lemeshow), 0.09 (Cox & Snell), 0.12 (Nagelkerke). Model χ^2^ =24.1, P < 0.001. B: coefficient of regression. SE: standard error; CI: confidence interval.

### Analysis of patients who survived 1 year post-transplantation

**Figure 2:**
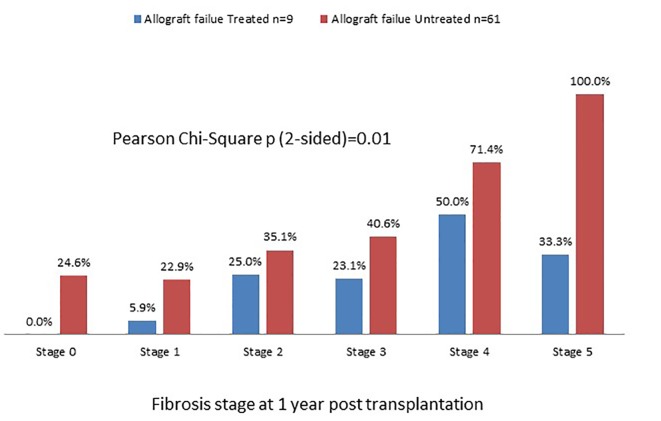
Graft survival rate in SVR and viremic patients categorized according to fibrosis stage at 1 year post-transplant. There is a trend of worsening graft failure from stage 0 to stage 5.

Among 252 transplant recipients studied, 226 (90%) were alive at 1 year: 116 with mild disease and 110 with severe disease. One hundred seventy-nine of the 226 (71%) patients received pegylated interferon and ribavirin with similar proportions in the two groups. Fifty-eight of the 179 patients treated (32%) were HCV RNA negative at 6 months following completion of treatment and thus achieved SVR with no difference between the two groups. We further categorized patients into those who had SVR versus those who remained viremic with or without therapy (Fig. 1). Figure 2 depicts distribution of allograft failure in the SVR and viremic groups according to stage of allograft fibrosis at 1 year post-transplant. In both groups, there was a trend towards higher graft loss with increasing fibrosis stage at 1 year post-transplant. Nevertheless, viral eradication significantly reduced the risk of allograft failure at all fibrosis stages. In a multivariable analysis, we evaluated the effect of baseline variables and fibrosis stage at 1 year on long-term graft survival (Table 7). Beyond the first year, recipient age and gender and use of T cell depletive therapy had no effect on graft survival; however, donor age continued to be highly predictive. Fibrosis stage 0, 1 or 2 had no influence but fibrosis stages 3/4 and 5/6 predicted inferior graft survival with hazard ratios of 2.25 and 3.46, respectively.

**Table 7:** Prediction of Graft Failure Beyond First Post-Transplant Year

Variable	B ± SE	Hazard ratio	P value
Fibrosis stage 5/6	1.24 ± 0.51	3.46	0.016
Fibrosis stage 3/4	0.81 ± 0.32	2.25	0.011
Fibrosis stage 2	0.54 ± 0.36	1.71	0.133
Recipient age	0.01 ± 0.02	1.01	0.649
Female gender	-0.13 ± 0.32	0.88	0.690
Donor age	0.03 ± 0.01	1.03	0.0007
Anti-T cell antibody	0.32 ± 0.30	1.38	0.285

Cox proportional hazards analysis. B: coefficient of regression. SE: standard error.

#### Long-term graft and patient survival

**Figure 3:**
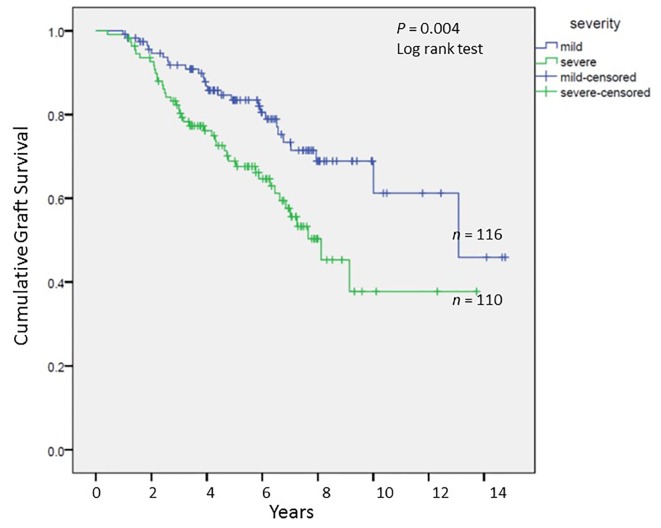
Cumulative graft survival among patients alive at 1 year post-transplant; Kaplan-Meier analysis, comparison by log-rank test.

**Figure 4:**
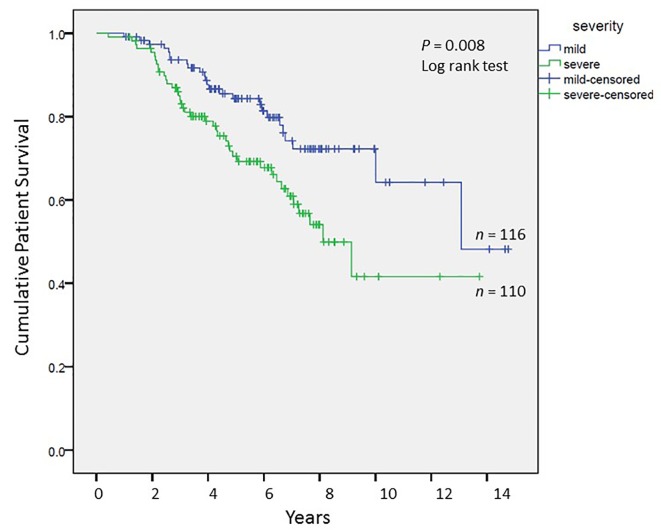
Cumulative patient survival among patients alive at 1 year post-transplant; Kaplan-Meier analysis, comparison by log-rank test.

**Figure 5:**
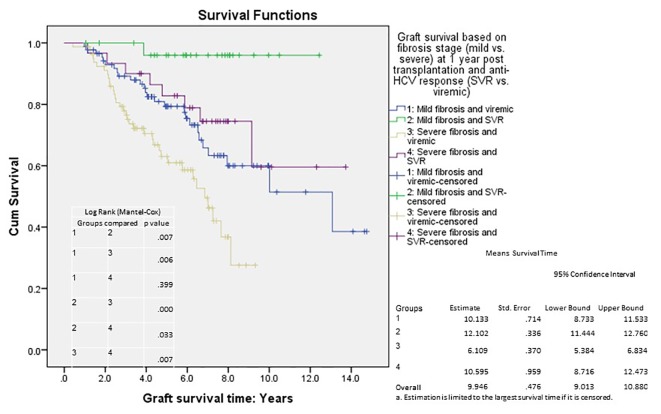
Cumulative graft survival according to fibrosis stage (at 1 year post-transplant) and treatment response; Kaplan-Meier analysis, comparison by log-rank test; mean survival with confidence intervals shown. Std. error: standard error.

We analyzed long-term graft and patient survival among patients alive at 1 year post-transplant, with recipients categorized to have mild versus severe disease as defined earlier (Fig. 3, 4). Among those alive at 1 year, the mean duration of graft survival was 9.9 (± 0.5) years, with 3-and 5-year survival of 92% and 84% in those with mild disease and 81% and 69% in those with severe disease (P = 0.004). The mean duration of patient survival was 10.3 (± 0.5) years, with 3-and 5-year survival of 94% and 84% in those with mild disease and 84% and 70% in those with severe disease (P = 0.008). We performed Cox hazard model and Kaplan-Meier analysis to evaluate impact of disease severity and viral eradication on graft survival (Table 8, Fig. 5). Compared to patients with mild disease who remained viremic, those with mild disease and SVR had significantly better graft survival whereas survival among those with severe disease and SVR was no different. Patients with severe disease who remained viremic had the worst graft survival of 63% at 5 years following transplant.

**Table 8:** Allograft Failure Risk Based on Treatment Status

	P value	HR	95.0% CI HR	
			Lower	Upper
Mild disease viremic	0.001			
Mild disease SVR	0.026	0.103	0.014	0.761
Severe disease viremic	0.007	2.050	1.218	3.451
Severe disease SVR	0.419	0.721	0.326	1.594

CI: confidence interval; HR: hazard ratio.

## Discussion

With recent advances in antiviral therapy, the outlook for liver allograft recipients with hepatitis C is expected to change. A significant proportion of recipients with HCV develop graft failure either due to severe cholestatic hepatitis or from rapid progression of fibrosis [13]. Viral eradication may alter disease course resulting in improved graft and patient survival. The risk of allograft cirrhosis among recipients who survive the first year post-transplant is about 25% over a period of 5 - 10 years, which suggests that the disease may remain indolent despite persistent infection [11]. Protocol liver biopsies may help identify patients at high risk of disease progression and graft failure [14]. Our study assessed the utility of allograft fibrosis stage at 1 year as an indication for antiviral therapy. Results indicated that patients with mild disease (stage 0 - 1) were at low risk of graft failure whereas those with severe disease (stage 2 - 6) were at higher risk of graft loss. On analyzing the effect of individual fibrosis stages, maximal effect leading to poor outcome was associated with stages 3 - 6 (Table 7).

All-oral interferon-free regimens are now the standard of care for hepatitis C [15]. However, prohibitive cost and limited availability in many parts of the world are barriers to their use [8, 16, 17]. Although not approved for use in transplant recipients, preliminary studies have shown high efficacy and tolerability of such regimens [5]. Transplant recipients constitute a special population that would derive high level of benefit from viral eradication; however, not all patients have severe disease. Thus, treatment may be reserved for patients at risk of rapid progression and graft failure to optimize use of limited financial resources. Conversely, recipients with mild disease could be carefully monitored without treatment until therapies become more affordable. In our current analysis, we attempted to identify recipients who would benefit from current treatment regimens versus those who could be followed without therapy.

We identified factors that predicted development of severe disease during the first post-transplant year. By univariable analyses, patients with mild versus severe disease differed in recipient gender, pre-transplant white cell count, donor age, acute rejection episodes, T cell depletive therapy, use of bolus corticosteroids and cumulative tarcolimus dose. By logistic regression analysis, recipient age and gender, donor age and use of anti-T cell antibody were independently predictive of severe disease. Most of those are well recognized predictive variables in HCV-positive recipients [11]. An interaction between gender and HCV status has been noted before. In an analysis of UNOS database, female recipients with HCV infection were noted to have significantly worse graft and patient survival compared to female recipients without HCV infection [1]. An effect of younger recipient age has not been noted before and therefore requires confirmation. One reason for this variance may be the endpoint in this part of our study that differed from other published reports: 1 year post-transplant versus cumulative long-term observation. Our study confirmed the deleterious effect of T cell depletive therapy in HCV-positive recipients possibly related to selection of resistant T-cell clones [4]. We also confirmed accelerated HCV disease course in older donor allografts that suggested an adverse effect of liver senescence [18, 19]. Still, it is difficult to include donor age in the transplant decision making process in view of the continued gap between organ availability and demand [20]. A policy to decline older organs for hepatitis C recipients may cause inordinate delays in transplanting such patients.

In conclusion, a significant proportion of liver transplant recipients with hepatitis C are at risk of allograft dysfunction and failure. Such patients could be identified by allograft biopsy at 1 year post-transplant. Recipients at high risk including those with FCH and those with moderate allograft fibrosis at 1 year post-transplant should be promptly initiated on antiviral therapy. Conversely, treatment may be deferred among recipients with mild disease until wider availability of more affordable and cost effective agents.

## Abbreviations

Anti-HBc: hepatitis B core antigen
CMV: cytomegalovirus
FCH: fibrosing cholestatic hepatitis
HBsAg: hepatitis B surface antigen
HCV: hepatitis C virus
HIV: human immunodeficiency virus
MELD: model for end-stage liver disease
SVR: sustained virologic response
